# College students and use of K2: an emerging drug of abuse in young persons

**DOI:** 10.1186/1747-597X-6-16

**Published:** 2011-07-11

**Authors:** Xingdi Hu, Brian A Primack, Tracey E Barnett, Robert L Cook

**Affiliations:** 1 Department of Epidemiology, University of Florida, Gainesville, Florida, USA; 2 Division of General Internal Medicine, Department of Medicine, University of Pittsburgh School of Medicine, Pittsburgh, Pennsylvania, USA; 3 Division of Adolescent Medicine, Department of Pediatrics, University of Pittsburgh School of Medicine, Pittsburgh, Pennsylvania, USA; 4 Department of Behavioral Science and Community Health, College of Public Health and Health Professions, University of Florida, Gainesville, Florida, USA; 5 College of Medicine, University of Florida, Gainesville, Florida, USA

## Abstract

**Background:**

K2 or "spice" has emerged as a popular legal alternative to marijuana among adolescents and young adults. However, no data has been published assessing prevalence of and associations with ever K2 use in any population. This study's aims were to examine prevalence of ever K2 use among a sample of college students, to determine characteristics of persons who use K2, and to access the association between K2 and other drug use.

**Findings:**

Ever use of K2 was reported by 69 (8%) of the sample of 852 college students. Response rate was 36%. Bivariate and multivariate analyses assessed whether sociodemographic characteristics and other drug use were associated with ever use of K2. Ever use of K2 was reported by 69 (8%) of the sample. Among these 69 individuals, 61 (88%) had used a cigarette and 25 (36%) had used a hookah to smoke K2. In multivariate analyses, K2 use was more common in males (vs. females, adjusted Odds Ratio (aOR) = 2.0, 95% Confidence Interval (CI) = 1.2-3.5, *p *= 0.01) and 1^st ^or 2^nd ^year college students (vs. 3^rd ^year or above, aOR = 2.4, 95% CI = 1.2-5.0, *p *= 0.02).

**Conclusions:**

Ever use of K2 in this sample was higher than ever use of many other drugs of abuse that are commonly monitored in adolescents and young adults. Although DEA had banned five synthetic cannabinoids recently, clinicians and public health officials concerned with substance abuse in youth should be aware of and monitor the use of this drug in college students over time.

## Findings

K2 or "spice" refers to a series of products that are advertised and sold legally as herbal blend incense. However, they are smoked by people to gain effects similar to marijuana, hashish, and other forms of cannabis. One or more synthetic cannabinoids, such as JWH-018, JWH-073 and CP 47, 497 C8 that mimic intoxication with marijuana with longer duration and poor detection on typical urine screens [1], are sprayed intentionally on dried herbs before they are packaged for sale as K2 [2]. These herbs have emerged as popular legal alternatives to marijuana among adolescents and young adults [3]. In response to the dangers of these products, on March 1, 2011, the Drug Enforcement Administration (DEA) issued the final order to temporarily ban five synthetic cannabinoids (JWH-018, JWH-073, JWH-200, CP 47,497 and CP 47,497 C8) following 18 states that had already implemented their own law or policy of controlling at one or more of these five synthetic cannabinoids [4].

According to the American Association of Poison Control Center (AAPCC), more than 2500 calls related to K2 were reported in 2010, compared with only 53 in 2009 [5]. Although no deaths have been reported, smoking K2 may produce several adverse health events, such as hallucinations, severe agitation, extremely elevated heart rate and blood pressure, coma, suicide attempts, and drug dependence, which is typically rare among classical cannabis users [6-8]. While some studies have detailed the adverse events and emergency department presentation associated with K2 use [8,9], it is unclear if this product is being used by persons who typically do not smoke, or if it is only being used by other smokers.

The primary objectives of this study are (1) to describe the prevalence of ever K2 use among a sample of college students and characteristics of persons who use K2; and (2) to assess associations between K2 and other drug use, including tobacco, cigarettes and marijuana.

In September 2010, University of Florida students were asked about K2 use as part of a study focusing on hookah tobacco smoking. These data were collected electronically via electronic mail invitations. Of 2396 emails delivered, 852 (36%) of students responded; this response rate is in the moderate-good range for this type of survey [10]. The institutional review boards of the University of Florida and the University of Pittsburgh approved this study.

Participants responded to items assessing sociodemographic data and tobacco smoking knowledge, attitudes, and behavior. Marijuana use was assessed by asking "have you ever smoked marijuana, even a puff?" K2 smoking was assessed using two items which asked "Have you ever smoked 'spice' (also known as 'K2' or 'legal weed') from a hookah?" and "Have you ever smoked 'spice' (also known as 'K2' or 'legal weed') from something other than hookah, such as a cigarette?" Two-way chi-squared analysis was performed to assess if sociodemographic data, tobacco and marijuana smoking were associated with K2 use. All sociodemographic variables that were significantly associated with ever K2 use in these bivariate analyses--and traditional risk factors for substance use, such as living arrangement and relationship--were included in a multivariate logistic regression model to assess the adjusted associations while controlling for other covariates. In order to avoid the potential for co-linearity, the multivariate model did not include both age and year in school. Instead, year in school was dichotomized to distinguish early college students (1^st ^or 2^nd ^year) from more advanced students. All analyses were performed using SAS statistical software, version 9.2.

The average (± SD) age of this sample of 852 college students was 20.6 ± 5.1 years. Nearly half (47%) of respondents were female. Racial/ethnic distributions were White (59%), Hispanic (17%), African American (8%), Asian/Pacific Islander (13%) and other (3%). Most students (70%) were in their first or second year, and 64% of participants were single at the time of data collection. The majority (71%) of surveyed students were living off-campus (Table 1), and ever use of other substances was reported by 34% for cigarettes, 36% for marijuana, and 39% for hookah tobacco.

**Table 1:** Associations between characteristics of college students and K2 use (N = 852)

Characteristic	Sample n (%)	K2 use n (row %)	***p*-value**^**a**^ **(Wald χ**^**2**^**, df)**	**aOR**^**b**^ (95% CI, *p *value)
Age				
18-19	572 (68)	55 (10)	**0.02 **(5.2, 1)	-
20+	265 (32)	13 (5)		-
Gender				
Female	397 (47)	22 (6)	**<0.01 **(6.9, 1)	1.0 (reference)
Male	452 (53)	47 (10)		2.0 (1.2-3.5, **0.01**)
Race				
White	492 (59)	43 (9)	0.43 (3.8, 4)	-
Hispanic	145 (17)	15 (10)		-
African American	66 (8)	2 (3)		-
Asian/Pacific Islander	107 (13)	7 (7)		-
Other^c^	25 (3)	2 (8)		-
Year in school				
3^rd ^year or above	252 (30)	11 (4)	**<0.01 **(6.9, 1)	1.0 (reference)
1^st ^/ 2^nd ^year	589 (70)	58 (10)		2.4 (1.2-5.0, **0.02**)
Relationship				
Married/Engaged^d^	296 (36)	17 (6)	0.06 (3.6, 1)	1.0 (reference)
Single	536 (64)	51 (10)		1.5 (0.8-2.6, 0.20)
Living arrangement				
On-campus^e^	243 (29)	22 (9)	0.32 (1.0, 1)	1.0 (reference)
Off-campus	596 (71)	45 (8)		1.2 (0.7-2.1, 0.49)
Cigarettes Ever				
Yes	288 (34)	53 (18)	**<0.01 **(61.1, 1)	-
No	555 (66)	16 (3)		-
Marijuana Ever				
Yes	294 (36)	63 (21)	**<0.01 **(106.6, 1)	-
No	535 (65)	5 (1)		-
Hookah Ever				
Yes	331 (39)	61 (18)	**<0.01 **(76.8, 1)	-
No	511 (61)	8 (2)		-

df, degree of freedom; aOR, adjusted Odds Ratio; CI, Confidence Interval

^a ^Chi-square analyses compare the proportion of those with and without K2 use for each sociodemographic characteristic (Wald χ^2 ^and degree of freedom are presented)

^b ^Multivariate logistic regression model includes gender, year in college, relationship, and living arrangements.

^c ^Respondents in "other" race group are Native American/Alaskan Native and multiracial or biracial, including Asian/Black or White/Black.

^d ^Married/Engaged indicates students who were married, divorced or engaged.

^e ^On-campus includes students living in campus residence hall, fraternity/sorority house and other university/college housing.

Ever use of K2 was reported by 69 (8%) of the sample. Among these 69 persons, K2 was used in a cigarette/joint by 61 (88%) and in a hookah by 25 (36%), respectively. Seventeen of the 69 individuals (25%) had used both hookahs and cigarettes to smoke K2. Among K2 users, ever use of other substances was reported by 61 (88%) for hookah tobacco, 63 (91%) for marijuana and 53 (77%) for cigarettes.

Bivariate analyses showed that ever use of K2 was significantly associated with age, gender, year in school, and ever use of other substances (hookah tobacco, cigarettes and marijuana smoking) but not race, living arrangement, or relationship (Table 1). Multivariate logistic regression models included all appropriate covariates except for age, which was highly correlated with year in school (Pearson correlation coefficient = 0.81). These analyses suggested that K2 use was more common among males compared with females and among 1^st ^or 2^nd ^year college students compared with 3^rd ^year or above college students (Table 1). Ever use of K2 was not significantly associated with living arrangement or relationship.

This study demonstrated that K2 or "spice", an emerging synthetic marijuana drug, had been used by 8% of a sample of college students from a large university; and that ever use of K2 was associated with male gender and younger age. To our knowledge, this is the first report of prevalence of ever K2 use and association of K2 use with sociodemographic characteristics and other substance use. Although 8% is below the prevalence of major substances, such as marijuana and tobacco, it is higher than the prevalence of many other drugs of abuse that are commonly monitored in college population, such as cocaine, LSD, heroin, sedatives, and anabolic steroids [11]. Considering the potential dangers of using K2 reported over the past several years, wider monitoring of this type of drug use would be warranted.

K2 could be a "gateway drug" related to other smoking substances. For example, students who smoked K2 might then advance to marijuana smoking. Although our data cannot show definitive temporal association, the data suggests that most users of K2 already smoked marijuana and therefore, it is not clear that K2 is a gateway drug to other smoking drugs. We found that K2 use was more common among 1^st ^and 2^nd ^year college students than 3^rd ^year college students. This may indicate that K2 use begins in high school, which would be consistent with case studies that emphasize use in secondary school [3,6,8]. However, it is also possible that they started to use this substance very early in college but then reduced its usage due to emerging legal alcohol use and/or social maturation. Longitudinal cohort data may help illuminate these uptake patterns.

This study was limited by its cross-sectional design. We cannot infer whether K2 use preceded or followed hookah tobacco use, for example. Additionally, outcome variables were assessed with self-report, rather than biochemical validation, and the sample was drawn from only one large university. In this study, an overall 36% response rate was obtained; this rate was 41% for females versus with 31% for males. This response rate is expected for this type of survey; in fact, systematic reviews have demonstrated 36% average response rates for 31 studies using email survey over 14 years [10,12]. Additionally, it is possible that non-response in this survey resulted in a more conservative estimate of K2 use in this population, because responses obtained from these types of surveys are generally higher from female, higher-achieving, less risk-taking sociodemographic groups [13].

In conclusion, we found that K2 had been used by nearly one in ten college students and was particularly common among males and early college students. Ever use of K2 was also higher than ever use of many other drugs of abuse that are commonly monitored in adolescents and young adults. Prevention of use of K2 or other synthetic cannabinoids products should be focused on male students or those who just enter college. Additionally, clinicians and public health officials concerned with substance abuse in youth should be aware of this drug and monitor its use in college students over time. The latest national ban of five synthetic cannabinoids does not necessarily indicate the end of K2 or "spice". For example, K2 manufacturers have already started to produce and sell a new generation of K2 products that are claimed to be "completely legal everywhere" (using a similar product with another, not yet illegal, synthetic cannabinoid). Future study with longitudinal data may be useful to investigate the impact of this new policy.

## Competing interests

The authors declare that they have no competing interests.

## Authors' contributions

XH performed the data analysis and wrote the manuscript. BAP, TEB and RLC participated in the design of the study, collected the data and helped to draft the manuscript. All authors read and approved the final manuscript.
